# [^18^F]FDG PET/CT value in the diagnostic evaluation of patients with polyarteritis nodosa

**DOI:** 10.1007/s00259-026-07857-8

**Published:** 2026-04-10

**Authors:** Nicolas Martinage, Julie Guyot, Gauthier Delaby, Damien Huglo, Emmanuel Ledoult, Clio Baillet

**Affiliations:** 1 Centre Hospitalier Universitaire de Lille, Nuclear Medicine Department, Lille University Hospital, Lille, France; 2 Internal Medicine and Clinical Immunology Department, Huriez Hospital, Centre de Référence des Maladies Auto-Immunes Systémiques Rares du Nord, Lille University Hospital, Méditerranée et Guadeloupe (CeRAINOM), Nord-Ouest, Lille, France

**Keywords:** Polyarteritis nodosa, FDG, PET/CT, Diagnosis, Ant-farm

## Abstract

**Purpose:**

Polyarteritis nodosa (PAN) is a rare necrotizing vasculitis of medium-sized vessels with heterogeneous presentations and a challenging diagnosis. While [^18^F]FDG PET/CT is established in large-vessel vasculitis, its role in PAN remains unclear. A distinctive “ant-farm” uptake pattern has recently been associated with PAN. This study assessed the diagnostic value of [^18^F]FDG PET/CT in PAN.

**Methods:**

Patients diagnosed with PAN who underwent [^18^F]FDG PET/CT between 2010 and 2025 were retrospectively included. Scans were blindly reviewed by two nuclear medicine physicians using a standardized grid. Abnormal uptakes, including the “ant-farm” pattern, were systematically recorded and correlated with clinical, biological, and histopathological data.

**Results:**

Forty-seven patients were included. Abnormal uptake was frequently found in the bone marrow (83%), lymph nodes (40%), and spleen (34%). Abnormal vascular uptake was observed in 36% of cases, predominantly in the femoral and tibial arteries. The “ant-farm” pattern was identified in 34% of patients with excellent inter-observer reproducibility (κ = 0.80). It was more frequent with recent PET/CT devices and whole-body acquisitions and was associated with higher C-reactive protein levels and fewer PAN-suggestive biopsies. Distinct uptake patterns were observed in etiologies of secondary PAN, including VEXAS syndrome.

**Conclusion:**

[^18^F]FDG PET/CT is a valuable diagnostic tool in PAN and may also help identify secondary causes. The consistent “ant-farm” pattern may help distinguish patients with more inflammatory features, negative biopsy results and less PAN-related organ involvement.

## Introduction

Polyarteritis nodosa (PAN) is a necrotizing vasculitis primarily affecting medium-sized vessels. Although its prevalence is low (approximately 31 cases per million inhabitants), the disease can lead to severe complications that may be fatal in some cases [1]. The clinical and biological manifestations of PAN are highly variable and may involve the skin, peripheral nervous system, or the uro-nephrological system (orchitis, renal failure). Cardiac and gastrointestinal involvements are less common but tend to be more severe. General symptoms such as fatigue, fever, and arthralgia/myalgia are frequently observed. Laboratory findings reveal an inflammatory syndrome characterized by marked elevation of C-reactive protein (CRP) and, occasionally, mild peripheral eosinophilia [2]. While PAN can occur as an idiopathic condition, some cases are associated with underlying disorders such as infectious diseases (hepatitis B virus (HBV) infection), hematologic disorders, solid tumors, or autoinflammatory syndromes (e.g., VEXAS syndrome). Consequently, PAN is classified into “primary” and “secondary” forms based on the presence of an associated disease [3]. The diagnosis of PAN relies on several criteria, including clinical features, histopathological examination, and the identification of arterial aneurysms. However, no specific diagnostic criteria exist, and biopsies can sometimes be inconclusive [4, 5]. Therefore, the diagnosis often remains challenging.

Due to its ability to detect vascular wall inflammation, [^18^F]FDG PET/CT has become an increasingly valuable imaging modality for diagnosing vasculitis. While [^18^F]FDG PET/CT is now a key tool in giant cell arteritis (GCA) [6], its role in medium-sized vessel vasculitis remains poorly documented. Several case reports and two case series [7–11] have described [^18^F]FDG PET/CT findings in adult patients with PAN, including increased uptake in the bone marrow, lymph nodes, and medium-sized vessels—most notably the femoral, humeral, and interosseous arteries. Additionally, a characteristic pattern of recurrent inter- and intramuscular FDG uptake has been reported in patients with PAN or lower limb vasculitis. This pattern, characterized by a combination of linear and patchy areas of increased [^18^F]FDG uptake, has been referred to as the “dirty muscle” or “ant-farm” appearance by some authors [9, 12, 13]. However, the relationship between this imaging pattern and PAN remains insufficiently understood.

The current study aims to assess the diagnostic value of [^18^F]FDG PET/CT in PAN by describing its metabolic imaging features and correlating them with clinical and paraclinical findings. Furthermore, this study examines whether [^18^F]FDG PET/CT may provide additional insight into the identification of secondary forms of the disease and underlying causes.

## Materials and methods

### Study population

This retrospective single-center study included patients treated in the internal medicine department at a tertiary referral center from January 2010 to January 2025. The patients presented features suggestive of polyarteritis nodosa (PAN) and underwent an [^18^F]FDG PET/CT following symptom onset. Diagnosis had to be established by the referring clinician and meet at least one of the following criteria retroactively: (i) biopsy-confirmed necrotizing vasculitis affecting medium-sized arteries, (ii) characteristic angiographic abnormalities, or (iii) a combination of clinical features consistent with medium-vessel systemic vasculitis, with other primary vasculitis excluded. Patients with organ-limited PAN were excluded. Additional exclusion criteria included positive ANCA serology, alternative diagnoses, missing data, or a diagnosis made before 2010. EL and JG individually reviewed all cases.

### [^18^F]FDG PET/CT analysis

Technical parameters and acquisition modalities were recorded. PET/CT technical details were described by the acquisition field (whole-body acquisition versus skull to mid-thigh acquisition without lower limbs) and by the PET/CT devices technology, categorized into three groups:

- *Digital PET/CT system*: Siemens Biograph Vision^®^, Philips Vereos^®^
- *Recent analog PET/CT systems*: Siemens Biograph mCT^®^, Horizon^®^ and GE Discovery^®^ (available after 2015)
- *Older-generation PET/CT system*: GE Discovery Rx^®^ (2009–2019)

All PET/CT scans were independently reviewed by two nuclear medicine physicians who were blinded to clinical data and each other’s interpretations (CB, GD). Disagreements were resolved by consensus; if unresolved, a third physician provided the final interpretation (DH). A standardized interpretation grid was used, pre-defined based on known and potential sites of involvement in PAN. Assessed features included: (i) Ant-farm pattern: linear and patchy uptake pattern as described in the literature [12] with SUVmax, anatomical distribution and visual intensity (either “mild” or “intense”); (ii) Vascular involvement: diffuse, homogeneous arterial hypermetabolism suggestive of systemic inflammation; focal uptake with vascular calcifications was excluded; visual intensity(“mild “/ “intense”), distribution location, and SUVmax were documented; (iii) Muscular involvement: considered pathological when focal (nodular) or diffuse and heterogeneous, excluding physiologic or tendon-related uptake; (iv) Articular involvement: considered potentially related to systemic inflammation when atypically located and without CT signs of degenerative disease or other local causes. (v) Lymph nodes involvement: pattern was considered as “pathological” if uptake was relatively intense (unequivocally higher than mediastinal uptake), located outside typical superficial reactive regions (e.g. axillary, inguinal, cervical), or associated with suspicious CT features (loss of fatty hilum, rounded shape); otherwise, uptake pattern was classified as “non-specific reactive” (vi) Testicular involvement: Defined by focal, asymmetric, or intense visual uptake; (vii) Bone marrow involvement: bone marrow uptake visually higher than hepatic uptake; distribution classified as limited to the spine or extending to the long bones diaphyses; (viii) Lung involvement : all the lung uptakes were reported and then classified as “inflammatory/infectious” when diffuse and associated with parenchymal infiltrates (consolidations and/or ground-glass opacities), and as “nodular” when focal and rounded; (ix) Gastrointestinal involvement : atypical hypermetabolism classified as “focal” or “diffuse”, excluding physiological uptake and patients receiving oral antidiabetic agents; (x) Pericardial/myocardial involvement : excluding non-specific myocardial [^18^F]FDG uptake (e.g. diffuse left ventricular or lateral wall uptake); (xi) Extra-articular cartilage involvement : including laryngeal, nasal and auricular cartilages; given the absence of physiological uptake in these sites, all were reported.

### Patient’s data collection

Clinical, biological, radiological, and, when available, histopathological data were collected. Biopsy sites were also recorded. PAN were classified as “secondary” if associated with underlying conditions known to be linked with PAN, such as hematologic disorders, solid tumors, chronic infectious diseases, or autoinflammatory disorders (notably VEXAS syndrome).

### Data analysis and correlation

All PET/CT findings were correlated with clinical and biological data that are consistent with areas of abnormal [^18^F]FDG uptake. Imaging findings were compared between primary and secondary PAN. Ant-farm uptake was analyzed in relation to clinical manifestations, biological abnormalities, histopathological findings, vascular hypermetabolism, and technical characteristics of the PET/CT devices.

### Statistical analysis

Statistical tests were performed using SPSS software (IBM^®^). Quantitative variables were presented as median values with corresponding first and third quartiles [Q1; Q3]. The significance level was set at α = 0.05. To limit α risk inflation, no statistical testing was conducted when the values being compared were reasonably close. The Mann-Whitney U test was used to compare quantitative variables. For categorical variables, either the Chi-squared test or Fisher’s exact test was applied. Inter-observer reproducibility was assessed using Cohen’s kappa (κ) coefficient.

## Results

### Population characteristics

#### Patients’ characteristics

One hundred twenty-four patients with suspected PAN were identified. Among them, 77 patients were excluded due to differential or uncertain diagnoses (*n* = 25). Thus, 47 patients were retrospectively analyzed (Table 1).

**Table 1:** Demographic, clinical, and paraclinical characteristics of the population

		*n* (%) / [Q1-Q3]
Demographic data	Sex (Male/Female) Age (median) [Q1; Q3]	36/11 (77/23) 67 [54.5; 72.5]
General symptoms	Overall health changes Fever	42 (89) 33 (70)
Musculoskeletal involvement	Myalgia Arthralgia	37 (79) 33 (70)
Cutaneous involvement		26 (55)
Neurologic involvement (multiple mononeuropathy)		11 (23)
Uro-nephrological involvement	Orchitis Renal failure	14 (30) 7 (15)
Cardiac involvement (myocarditis)		9 (19)
Digestive involvement (pain, bleeding, perforation)		8 (17)
Corticosteroid or immunosuppressive treatment at the time of PET/CT		3 (6)
Laboratory findings	CRP (median) [Q1 – Q3] MCV (median) [Q1 – Q3] Monocytosis Anemia	160 [95.5–265] 89,6 [84.8–92,1] 26 (55) 38 (88)
Arterial aneurysm		11 (23)
Pathology	Biopsy sample Histological features of PAN	40 (85) 23 (49)
Secondary PAN - HBV - Other chronic infection (dental, aspergillosis) - Solid cancer - Hemopathy (MDS; CMML; HL; NHL) - CHIP mutation - VEXAS syndrome - ADA2 deficiency - Inflammatory bowel disease		26 (55) 1 4 1 8 (5; 1; 1; 1) 9 2 1 2

*MDS *Myelodysplastic syndrome, *CMML* Chronic myelomonocytic leukemia, *HL* Hodgkin lymphoma, *NHL* Non-Hodgkin lymphoma, *CHIP* Clonal hematopoiesis of indeterminate potential

The median age of the population was 67 years old, with a male predominance (77%). The most common clinical symptoms included general conditions such as overall health changes (89%) and fever (70%), as well as musculoskeletal issues such as myalgia (79%) and arthralgia (70%). Cutaneous involvement was observed in 55% of cases, and neurological involvement in 23%. Three patients (6%) were receiving corticosteroid and/or immunosuppressive therapy at the time of PET/CT. The corresponding regimens were as follows: low-dose oral corticosteroids (5 mg/day) (*case 12*), azathioprine 50 mg/day combined with oral corticosteroids (12.5 mg/day) (*case 17*), and oral corticosteroids alone (17.5 mg/day) (*case 47*). The median CRP value was 160 mg/L. Forty patients (85%) underwent a biopsy, with pathological findings suggestive of PAN in 23 (49%). Twenty-six patients (55%) had a secondary form of PAN, including HBV infection (*n* = 1), VEXAS syndrome (*n* = 2), and ADA2 deficiency (*n* = 1). 

#### Technical PET/CT characteristics

Of the 47 PET/CT, 33 (70%) were performed with recent devices. Among these, 6 used digital PET/CT (4 Vision Siemens^®^, 2 Vereos Philips^®^), while 27 were analog PET/CT (21 mCT Siemens^®^, 5 Discovery GE Healthcare^®^, 1 Horizon Siemens^®^). Fourteen scans (30%) involved PET/CT Discovery Rx GE Healthcare^®^. Twenty-five patients (53%) underwent whole-body acquisition. All upper limbs were in the acquisition field for 13 (28%) PET/CT scans. The median time between radiotracer injection and acquisition was 63.0 min [59.0; 70.5], and the median blood sugar level was 1.03 g/L [0.90; 1.12].

### FDG PET/CT findings

#### General PET/CT findings

General [^18^F]FDG PET/CT findings are summarized in Table 2. Abnormal bone marrow uptake was detected in 39 patients (83%), with a median SUVmax of 4.3 and diaphyseal extension in 10 patients. The median CRP was significantly higher in patients with abnormal bone marrow uptake compared to those without (178 vs. 52 mg/L). Four patients had CRP < 80 mg/L, including two with myelodysplastic syndrome (MDS).

**Table 2:** PET/CT findings with main clinical and paraclinical significance

	Abnormal FDG uptake (%)	Clinical and laboratory features
Bone marrow	39 (83)	35 patients with CRP > 80 mg/L
- extended to long bones diaphysis	10	6 clonal hematologic disorders
Lymph node	19 (40)	
- non-specific reactive pattern	11	
- pathological involvement pattern	8	1 VEXAS, 1 HL, 1 CMML
Spleen	16 (34)	2 MDS, 1 CMML, 1 VEXAS
Muscle	10 (21)	7 myalgia
Inflammatory pattern	8 (17)	
- multifocal nodular	3	1 MDS, 1 VEXAS
- diffuse heterogeneous	5	
Gastrointestinal	9 (19)	*NA*
- focal pattern	7	3 abdominal pains
- diffuse uptake	2	1 IBD, 1 abdominal pain
Lung	7 (15)	
- infectious/inflammatory pattern	6	
- nodular uptake	1	
Testis	5 (11)	No clinical orchitis
Pericardial/myocardial	4 (9)	2 myocarditis
Extra-articular cartilage	3 (6)	2 VEXAS

*HL* Hodgkin lymphoma, *CMML* Chronic myelomonocytic leukemia, *MDS* Myelodysplastic syndrome, *IBD* Inflammatory bowel disease, Clonal hematologic disorder includes MDS and CMML; *NA* not applicable

Abnormal lymph node uptake was detected in 19 patients (40%), mainly with nonspecific reactive pattern (*n* = 11). Among the 8 patients with features suggestive of pathological involvement, 2 had hematologic malignancies (Hodgkin lymphoma, CMML), 1 had VEXAS syndrome, and 3 carried CHIP mutations. Uptake occurred both above and below the diaphragm without a preferential distribution. Abnormal splenic uptake occurred in 16 patients (34%), mostly homogeneous (*n* = 14), with a median splenic length of 8.7 cm.

Abnormal muscular uptake was observed in 10 patients (21%), with features indicative of inflammation in 8 (5 nodular, including 1 MDS and 1 VEXAS, and 3 heterogeneous). It was associated with myalgia in 7 patients.

Abnormal digestive uptake was found in 9 patients (19%), with focal uptake in 7 (including 3 with abdominal pain), and diffuse uptake in 2 (located in the gastric fundus and terminal ileum). Testicular hypermetabolism was noted in 5 males (14%), pulmonary uptake in 7 (15%), and myo/pericardial involvement in 4 (9%) (including 2 with pericardial effusion, 1 with hypermetabolic mass).

Cutaneous or subcutaneous uptake was observed in 4 patients (9%), with 3 presenting lower limb infiltrates and 1 showing multiple nodules. Articular uptake was seen in 3 patients (6%), including bilateral temporomandibular, peripheral joints, and the cervical interspinous region. Extra-articular cartilaginous uptake was also noted in 3 patients (6%), mainly in the nasal bridge (*n* = 2) and ear (*n* = 1). One patient exhibited bilateral diffuse renal cortical uptake without dysfunction. Focal dental uptake suggestive of infection was identified in 4 cases, confirmed by CT in 3. Additionally, eleven CT-detected dental lesions showed no abnormal uptake on PET/CT.

#### Vascular uptakes

**Fig. 1:**
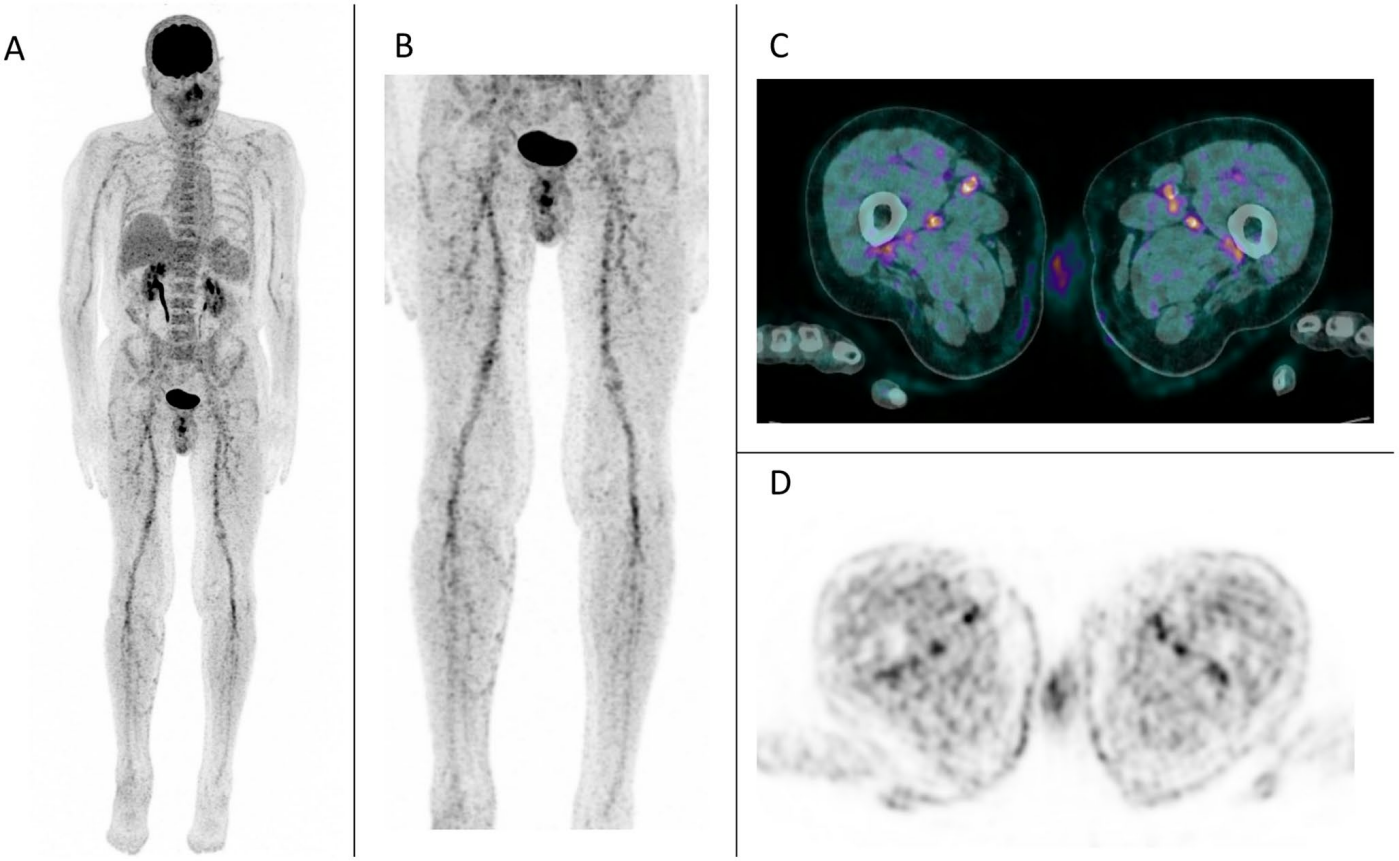
Intense vascular uptake in the femoral arteries with unusual visibility of distal branches and mild uptake in the right tibio-peroneal arteries. *Case 44* – (**A**) Maximum Intensity Projection (MIP); **B** MIP centered on thighs and legs; **C** axial fused PET/CT and (**D**) axial PET-only images at the level of the thighs

Vascular FDG uptake suggestive of inflammation was observed in 17 patients (36%) (Fig. 1*– case* *44*; Table 3). Uptake was considered equivocal in 3 cases and was ultimately classified as negative. Vascular hypermetabolism predominantly involved branches of the femoral (*n* = 16; 94%), anterior tibial and tibioperoneal (*n* = 12; 71%), humeral (*n* = 9; 52%), and internal iliac (*n* = 4; 24%) arteries. Less common sites included the common carotid, axillary branches, celiac trunk, and external iliac arteries (each *n* = 1).

**Table 3:** Abnormal PET/CT vascular uptake characteristics

PET/CT vascular uptake suggestive of inflammatory involvement (%)	17 (36)
Sites (%)	
- femoral artery and branches	16 (94)
- tibial anterior – tibioperoneal arteries and branches	12 (71)
- humeral artery	9 (52)
- internal iliac artery	4 (24)
- other	4 (24)
Visual intensity and extent (%)	
- intense	6 (35)
- mild	11 (65)
Median SUVmax [Q1 ; Q3]	3.7 [3.3; 4.7]
Inter-observer agreement (κ)	0.216

The median SUVmax was 3.7 [3.3; 4.7]. Uptake was visually mild and limited in 11 patients (median SUVmax : 3.6 [3.4;4.7]) and more intense in 6 (median SUVmax: 3.9 [3.2;5.4]).

Among the 17 patients with vascular uptake, 14 (82%) underwent biopsies, with histopathologic evidence of medium-sized vessel vasculitis in only 1 case (7%). Meanwhile, 22 of 26 (85%) patients without vascular uptake showed suggestive histological findings.

Discrepancies in vascular uptake interpretation occurred in 14/47 cases (30%), all of which were ultimately considered pathological. Interobserver agreement for vascular findings was low (κ = 0.216; *p* = 0.121). 

#### Ant-farm pattern uptakes

**Fig. 2:**
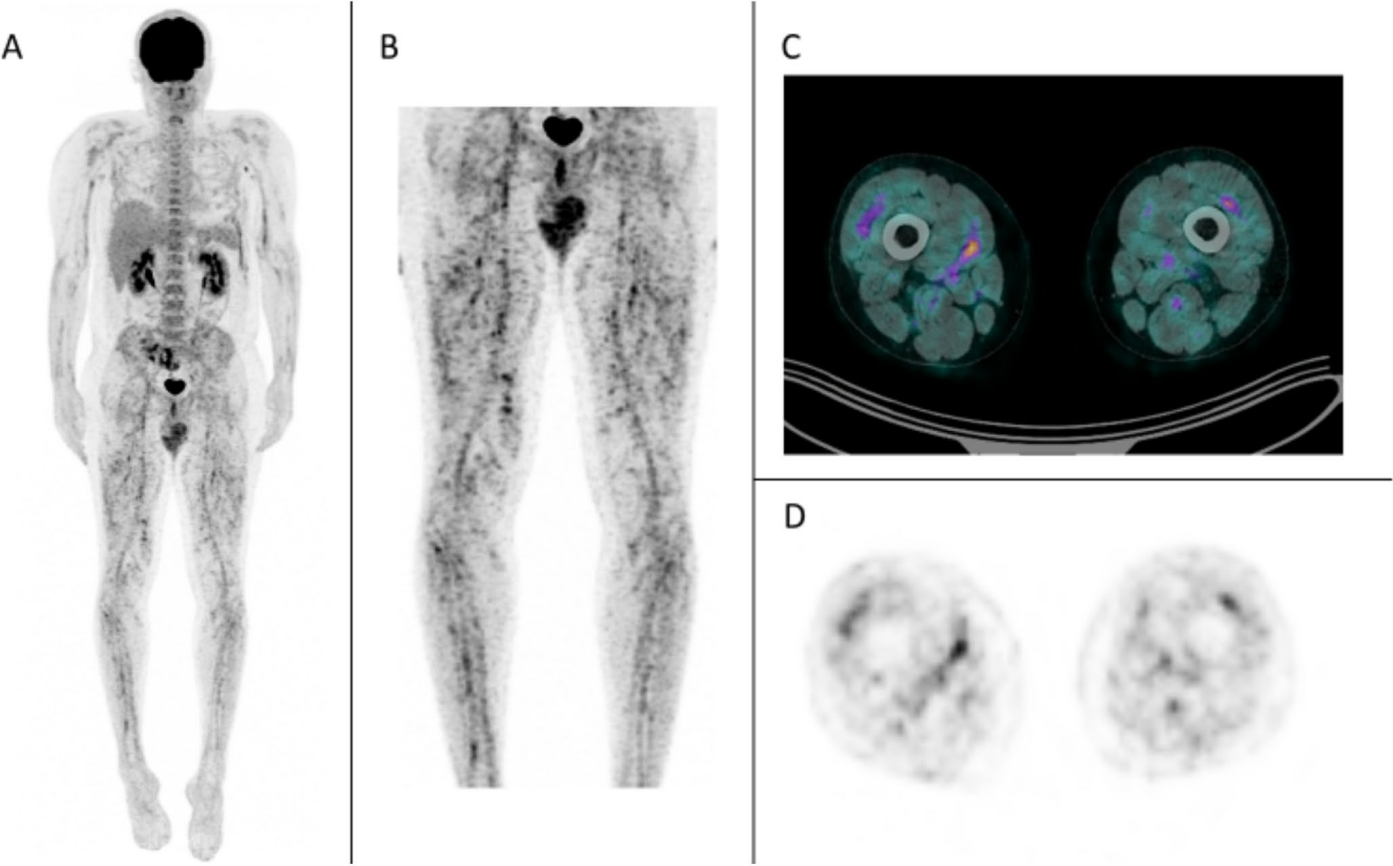
Ant-farm intense uptake pattern in lower limbs. Case 2; **A** Maximum Intensity Projection (MIP); **B** MIP centered on the thighs and legs; **C** axial fused PET/CT and (**D**) axial PET-only images at the level of the thighs. The findings show heterogeneous linear hypermetabolism along major vascular branches and within intermuscular spaces, associated with more diffuse punctate areas of increased uptake. The overall pattern appears "dirty" or "ant-farm-like," best visualized on the MIP

Sixteen patients (34%) exhibited a mixed patchy and linear ant-farm uptake pattern (Fig. 2***–*** *case 2*; Table 4), involving the lower limbs in all cases—thighs in 14 (88%) and legs in 12 (75%). Arm involvement was rare (*n* = 2; 13%). Two equivocal cases were ultimately considered negative. Uptake was visually intense in 4 patients and mild in 12. Median SUVmax was 3.7 [2.5; 4.4], ranging from 2.4 to 6.1. Discrepant interpretations occurred in 5 cases, with a high interobserver agreement (κ = 0.804, *p* < 0.001).

**Table 4:** PET/CT Ant-farm uptake characteristics

PET/CT Ant-farm uptake (%)	16 (34)
Lower limbs (%)	16 (100)
Upper limbs (%)	2 (13)
Median SUVmax [Q1 – Q3]	3.7 [2.5 ; 4.4]
Visual intensity and extent (%)	
- intense	12 (75)
- mild	4 (25)
Inter-observer agreement (κ)	0.804

### Ant-farm pattern distribution based on technical, clinical, and paraclinical features

#### Technical PET/CT characteristics

**Fig. 3:**
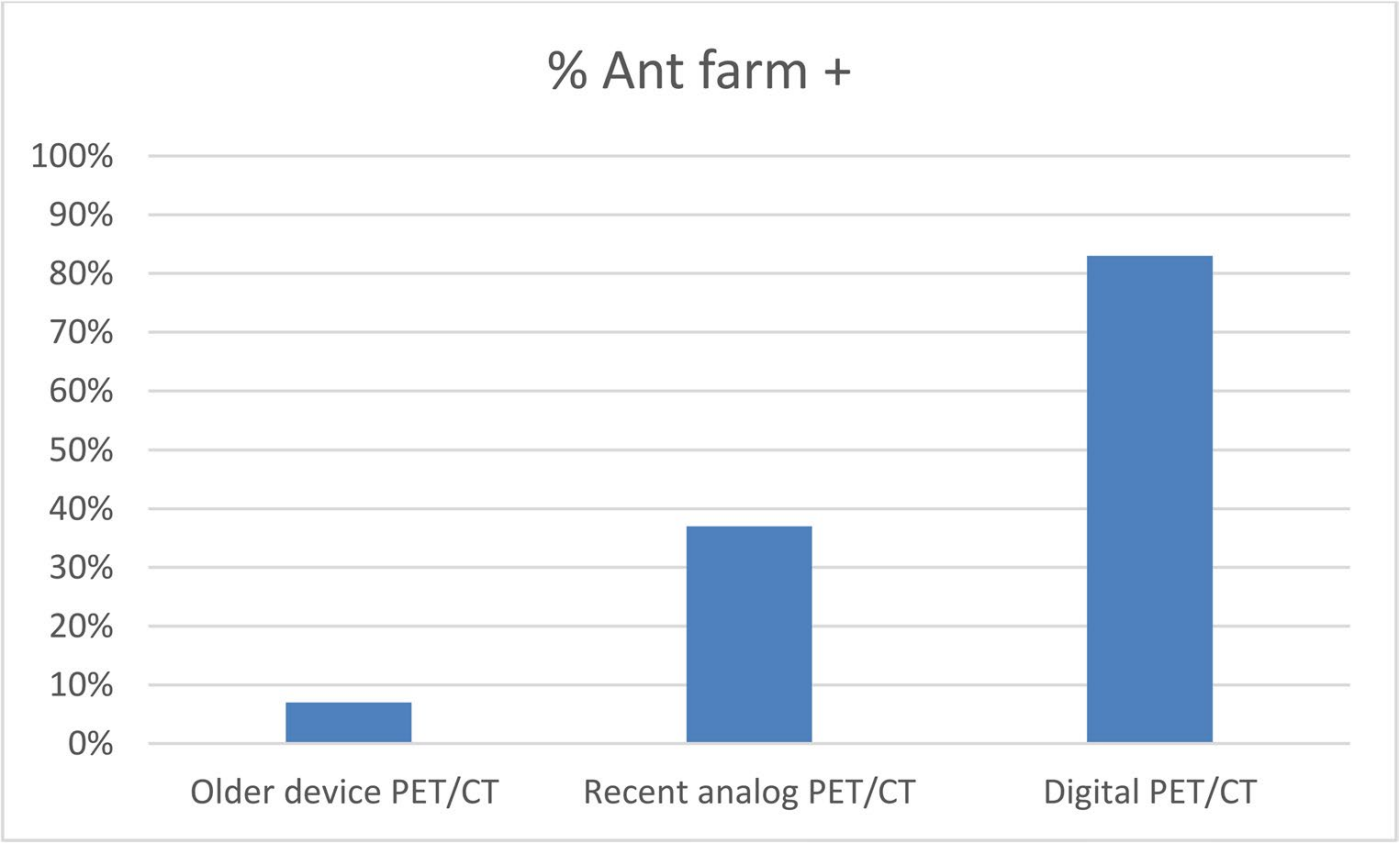
Distribution of ant-farm pattern uptake according to PET/CT generations. Older PET/CT: 7% (*n* = 1/14); Recent analog PET/CT: 37% (*n* = 10/27); Digital PET/CT: 83% (*n* = 5/6)

The ant-farm uptake pattern was significantly more frequent in patients who underwent whole-body PET/CT, including the lower limbs (*n* = 14/26; 56% vs. *n* = 2/24; 9%; *p* = 0.001) and with newer-generation scans (*n* = 15/33; 45% vs. *n* = 1/14; 6%; *p* = 0.011), especially digital PET/CT. However, whole-body acquisitions were more commonly performed with recent devices (*n* = 2/14, *n* = 18/27, *n* = 5/6 for older device, recent analog and digital PET/CT respectively) (Fig. 3). Two patients with ant-farm uptake did not have lower-limb acquisition; both scans were performed on more recent systems.

In analyses restricted to whole-body acquisition, the ant-farm sign was also more frequent on digital PET/CT than on older analog devices (80% vs. 50% respectively).

#### Clinical manifestations

**A**lthough not statistically significant, patients with ant-farm uptake tended to present more frequent systemic symptoms (such as overall health changes, myalgia, and fever). In contrast, patients without this uptake pattern tended to have higher rates of cutaneous (65% vs. 38%), neurological (29% vs. 13%), and myocardial (26% vs. 6%) involvements (Table 5). None of the patients receiving corticosteroid and/or immunosuppressive therapy at the time of PET/CT exhibited an ant-farm pattern (*n* = 3).

**Table 5:** Clinical, Biological, and Histopathological Characteristics According to Ant-Farm Uptake

Clinical and vascular involvement	Ant-farm (+) (%) (*n* = 16)	Ant-farm (–) (%) (*n* = 31)	*p*-value
Overall health changes	16 (100)	26 (84)	*N.S.*
Myalgia	15 (88)	23 (74)	*N.S.*
Fever	12 (71)	21 (68)	*N.S.*
Arthralgia	10 (59)	23 (74)	*N.S.*
Orchitis	5 (38) $	9 (39) $	*N.S.*
Cutaneous involvement	6 (38)	20 (65)	*N.S.*
Digestive involvement	3 (19)	5 (16)	*N.S.*
Neurological involvement	2 (13)	9 (29)	*N.S.*
Myocarditis	1 (6)	8 (26)	*N.S.*
Vascular uptake	14 (88)	3 (10)	< 0.001
Biological findings	Ant-farm (+) (*n* = 16)	Ant-farm (-) (*n* = 31)	*p*-value
CRP (mg/L) (median) [Q1; Q3]	231 [175; 301.3]	134 [51.5; 213]	0,008
MCV (fl.) (median) [Q1; Q3]	89 [85; 90.4]	90.8 [81.6; 92.7]	*N.S.*
Eosinophil count (/mm^3^) (median) [Q1; Q3]	150 [75; 375]	100 [100; 300]	*N.S.*
Monocytosis (%)	11 (69)	15 (44)	*N.S.*
Anaemia (%)	13 (81)	25 (81)	*N.S.*
Pathology findings	Ant-farm (+) with biopsy (*n* = 14; 88%)	Ant-farm (-) with biopsy (*n* = 26; 84%)	*p*-value
Histopathology suggestive of PAN (%)	1 (7)	22 (85)	< 0.001
-muscle biopsy (%) -skin biopsy (%) -alternative biopsy site (%)	11 (79) 2 (14) 1 (7)	8 (31) 17 (65) 1 (4)	

Ant-farm (+) / (-) patients with and without ant-farm uptake respectively; $ percentage of male patients with and without ant-farm uptake, respectively; NS not significant

#### Histopathological findings

Biopsy rates were similar at 88% and 84% for the ant-farm and non-ant-farm patients, respectively. In the ant-farm group, muscle was the most common biopsy site (79%), while skin biopsies were predominant in the non-ant-farm group (65%). Among those who underwent biopsy, only one patient (7%) in the ant-farm group had histological findings suggestive of PAN, compared with 22 patients (85%) in the other group, including 16 skin, 5 muscle, and 1 liver biopsies (Table 5). This difference reached statistical significance (*p* < 0.001).

#### Biological findings

The median CRP was significantly higher in patients with ant-farm uptake (231 vs. 134 mg/L; *p* = 0.008). No significant differences were observed for other biological parameters (Table 5).

#### Vascular findings

Vascular uptake on PET/CT was significantly more frequent in patients with ant-farm uptake (88% vs. 10%; *p* < 0.001). Conversely, 82% of patients with vascular uptake also showed ant-farm pattern, versus only 7% among those without vascular involvement. Aneurysms on conventional imaging were more frequent in the non-ant-farm group (29% vs. 12%).

Analyses restricted to primary PAN cases yielded similar results.

### FDG PET/CT findings according to primary/secondary PAN status

#### Primary versus secondary PAN

Comparison between primary and secondary PAN revealed that patients with secondary disease exhibited a higher frequency of lymph node uptake (*n* = 14; 54% vs. *n* = 5 ; 24%), bone marrow uptake extended to long bones (*n* = 9/22 ; 41% vs. *n* = 1/16 ; 6%), focal dental hypermetabolism (*n* = 4 ; 15% vs. *n* = 0), and joint uptake indicative of inflammatory involvement (*n* = 3 ; 12% vs. *n* = 0). Patients with primary PAN demonstrated more frequent vascular uptake (*n* = 10; 48% vs. *n* = 7; 27%) and ant-farm uptake (10 patients, 47% vs. 6 patients, 23%). None of the observed hypermetabolic patterns reached statistical significance between the two groups.

Within the cohort, 10 patients (21%) demonstrated bone marrow uptake with atypical extension to long bones. Among them, 6 had hematologic disorders (3 MDS, 2 MDS associated with VEXAS syndrome and 1 CMML). Among the 8 patients diagnosed with a myeloid neoplasm, 6 (75%) exhibited this specific bone marrow uptake pattern. 

#### VEXAS syndrome associated with PAN

**Fig. 4:**
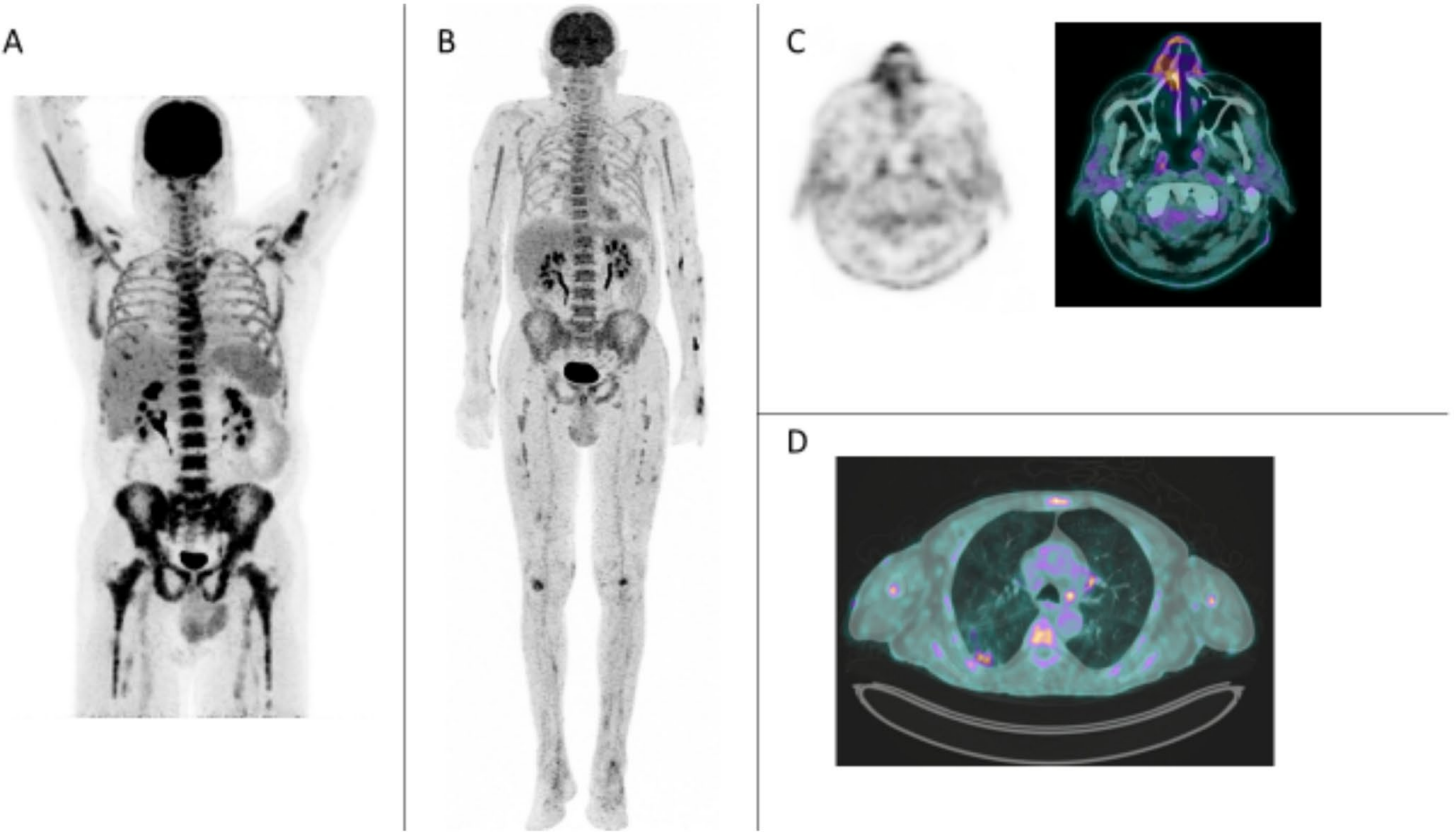
Metabolic involvement in VEXAS syndrome associated with PAN. MIP views of *case 41* (**A**) and *case 40* (**B**); diffuse bone marrow hypermetabolism extending to the diaphysis of long bones (**A**, **B**), multiple hypermetabolic muscular and cutaneous nodules (**B**), hypermetabolism of the nasal cartilage (**C** – *case 41*) and hypermetabolic pulmonary infiltrate (**D** – *case 40*).

Two patients were diagnosed with VEXAS syndrome (Fig. 4 - *cases 40* and *41*). Both showed osteomedullary FDG uptake extending into the long bone diaphyses (SUVmax 9.8 and 5.2) and nasal cartilage hypermetabolism (SUVmax 4.1 and 4.8). Pulmonary uptake was present in both patients: bilateral subpleural infiltrates (case 40) and interstitial involvement (case 41). Histopathology suggested PAN in case 40, whose PET/CT revealed diffuse multinodular muscular and subcutaneous uptake (“tiger man” pattern), FDG-avid cervical lymphadenopathy, and hypermetabolic thyroiditis. Case 41 demonstrated bilateral temporomandibular and homogeneous splenic uptake. Clinically, both had systemic inflammation with cutaneous manifestations. Case 41 also exhibited arthromyalgias and orchitis.

## Discussion

Among this cohort of 47 patients with PAN who underwent [^18^F]FDG PET/CT, 34% showed an ant-farm uptake pattern, consistently involving the lower limbs, whereas upper-limb involvement was rare (13%). This pattern was associated with significantly higher CRP levels and fewer PAN-positive biopsies. Inflammatory vascular uptake was observed in 36% of patients, mainly affecting the femoral arteries and their branches (94%) and showing mild visual intensity.

The most frequent sites of hypermetabolism were bone marrow (83%), lymph nodes (40%), and spleen (34%). Osteomedullary uptake extending to long bones was seen in 75% of patients with myeloid neoplasms, and both cases of VEXAS syndrome exhibited nasal cartilage involvement.

### Ant-farm uptake

This cohort shows that the mixed patchy and linear ant-farm uptake pattern is a common and consistent finding on [^18^F]FDG PET/CT in patients with PAN.

The underlying mechanism of this PET pattern remains unclear. However, it can be hypothesized that it reflects inflammation of medium-sized vessels—either perivascular or vascular wall inflammation. Indeed, this hypermetabolic activity is mainly seen in intermuscular areas, which consist of vascularized connective tissues. Some authors have suggested that linear uptakes indicate inflammation of medium- or large-sized vessels, while patchy uptakes may be linked to small-vessel vasculitis [12]. Furthermore, in this study, 82% of patients with vascular hypermetabolism also showed an ant-farm pattern. In some cases, it was challenging to clearly differentiate the ant-farm pattern from isolated vascular uptake. These findings suggest that the ant-farm and vascular uptake patterns may represent a continuum rather than two distinct entities.

This study enhanced understanding of the diagnostic importance of this imaging pattern. Interestingly, almost all patients with the ant-farm uptake showed no histopathology findings suggestive of PAN. This observation was also reported in the Mayo clinic cohort [11] (abnormal linear muscular uptake similar to the ant-farm pattern reported in 6 patients with only a single case of positive biopsy). The only “ant-farm patient” in our cohort with a biopsy suggestive of PAN was found to have ADA2 deficiency, a rare monogenic autoinflammatory syndrome.

Furthermore, patients with the ant-farm pattern appear to exhibit a distinct phenotype, characterized by higher CRP levels (*p* = 0.008), more systemic symptoms, and less cutaneous, neurological, or myocardial involvement.

Thus, ant-farm uptake appears to preferentially occur in patients with inflammatory features caused by medium-sized vessel vasculitis, but not in those with only organ damage due to ischemic vasculitis. The limited role of biopsy might relate to the size of the vessels involved (0.5 to 2.0 mm in the case of medium-sized vessels and their initial branches), which are generally outside the range sampled by standard (muscle) biopsies. These typically only capture smaller-caliber vessels (i.e., last subdivision) below 0.4 mm.

In light of these observations, the specificity of the ant-farm pattern for PAN remains uncertain. While this particular muscular and vascular uptake is described in systemic or lower-limb-limited PAN, sometimes referred to under different names (e.g. ‘dirty muscle’ or ‘Wagyu sign’) [7, 9, 12, 14], similar patterns have also been reported in giant cell arteritis (GCA) or cryoglobulinemic vasculitis [11, 15]. Therefore, it cannot be ruled out that the ant-farm pattern may be associated with a condition other than PAN, such as an overlap syndrome with another vasculitis. The optimal way to assess the specificity of the ant-farm sign would be to estimate its prevalence in a large cohort of patients with PAN and other forms of vasculitis. However, such a study would be particularly challenging to conduct, owing both to the low prevalence of the ant-farm sign and to the difficulty in standardizing the selection criteria (e.g. type of vasculitis, unexplained inflammatory syndromes, or alternative conditions). Despite its uncertain specificity, the ant-farm sign may represent a useful diagnostic clue suggestive of an ‘‘inflammatory’’ form of medium-vessel vasculitis, including PAN, particularly when conventional tests, such as biopsy, are negative.

This study also shows that ant-farm uptake is consistently observed in the lower limbs and is more frequently seen in digital PET/CT. This underscores the importance of including the lower limbs in the acquisition field when evaluating suspected PAN. When analyses were restricted to whole-body acquisitions, the ant-farm sign remained more frequent on digital PET/CT than on older analog systems although the difference was smaller. These findings suggest that this higher detection rate may be related to both more frequent whole-body acquisitions and improved contrast recovery and spatial resolution, enabling better detection of small-vessel abnormalities. Accordingly, given the heterogeneity of PET/CT technologies, nuclear medicine physicians should be aware of this potential technical limitation when interpreting [^18^F]FDG PET/CT in the setting of vasculitis.

### Other 18FDG PET/CT uptake

Vascular FDG uptake was observed in over a third of patients in our cohort, supporting the diagnostic value of PET/CT in evaluating suspected vasculitis. They align with previously published data [7, 11]. Inter-observer agreement for vascular involvement was low and not significant (κ = 0.216). Upon reviewing discordant cases, FDG uptake intensity appeared to be an unreliable criterion. Comparison with liver SUVmax, as used in GCA [16], is not applicable to smaller vessels due to their inherently low uptake. The extent of vascular uptake, particularly the unusual visibility of distal vessel branches, seems to be a more reliable indicator. This “tree-root-like” appearance has been previously described in various types of vasculitis, including ANCA-associated vasculitis [17]. These results underscore the need for standardized [^18^F]FDG PET/CT interpretation criteria for medium-sized vessel vasculitis.

Osteomedullary hypermetabolism was the most frequent metabolic finding, as previously reported [7]. In most cases, it was related to reactive bone marrow, due to systemic inflammation, as suggested by higher CRP levels in these patients. In some cases, this osteomedullary hypermetabolism extended to long bones. These patients often presented with underlying hematologic disorders, such as myeloid malignancies. Therefore, our findings suggest that [^18^F]FDG PET/CT may be useful in detecting secondary forms of PAN related to hematological malignancies. Although PET/CT has lower sensitivity than dental CT, some focal dental uptakes of infectious origin were detected. These findings are important to note, as PAN may be triggered by infection and can fully recover following adequate dental management [18–19]. Finally, other FDG-uptake sites were poorly correlated with clinical or biological involvement.

### Primary versus secondary PAN

In addition to atypical osteomedullary hypermetabolism, patients with VEXAS syndrome appear to exhibit a distinct FDG uptake pattern, including nasal cartilage and pulmonary infiltrate uptake, and long-bone osteomedullary hypermetabolism—potentially related to underlying myelodysplasia. These findings are consistent with previously reported series of [^18^F]FDG PET/CT in VEXAS syndrome [20], and correlate with the known clinical manifestations of the disease. Consequently, VEXAS syndrome should be considered as the underlying diagnosis when a patient with suspected PAN demonstrates this particular FDG uptake pattern. No other significant hypermetabolic findings were consistently associated with either primary or secondary forms of PAN.

### Limitations

The first limitation of this study is its retrospective design. However, given the low incidence of PAN, a prospective approach is difficult to achieve for this type of investigation. Second, only patients who underwent [ ^18^ F]FDG PET/CT were included. Therefore, we cannot exclude a potential selection bias toward a specific PAN profile, possibly more atypical and/or severe. Although the overall distribution of organ involvement in our cohort is broadly consistent with that reported in other large PAN series [2, 18], some differences were observed (e.g. older patients, higher prevalence of myocarditis, and lower prevalence of mononeuritis multiplex). Finally, [^18^F]FDG PET/CT examinations were performed over a wide time period. Consequently, we cannot exclude that the interpretation of some parameters may have been influenced by scanner generation. Nevertheless, except for the ant-farm sign and vascular uptake (which were more frequently observed on digital PET/CT systems), the assessment of most other organ involvements is unlikely to have been substantially affected by PET/CT system generation.

## Conclusion

[^18^F]FDG PET/CT appears to be a valuable diagnostic tool for polyarteritis nodosa (PAN), with an uptake pattern (“ant-farm”), that is highly reproducible and may help identify a specific inflammatory phenotype among patients with diagnostically challenging vasculitis and negative biopsy results. This pattern might become more commonly described in the future as digital PET-CT devices improve contrast and spatial resolution. Additionally, some FDG uptake patterns may indicate underlying causes of secondary PAN, such as VEXAS syndrome or hematological malignancies.

## Data Availability

The datasets generated and analysed during the current study are available from the corresponding author on reasonable request, in accordance with institutional and data protection regulations.
